# Pubococcygeal Sling versus Refixation of the Pubocervical Fascia in Vesicovaginal Fistula Repair: A Retrospective Review

**DOI:** 10.1155/2018/6396387

**Published:** 2018-10-31

**Authors:** Rachel Pope, Prakash Ganesh, Jeffrey Wilkinson

**Affiliations:** ^1^Baylor College of Medicine, Department of Obstetrics and Gynecology, Division of Global Women's Health, 6651 Main St., Houston, TX 77030, USA; ^2^International Training and Education Center for Health (I-TECH), University of Washington, Lilongwe, Malawi

## Abstract

Urethral incontinence is an issue for approximately 10–15% of women with an obstetric fistula. Various surgical interventions to prevent this exist, including the pubococcygeal sling and refixation of the pubocervical fascia. Neither has been evaluated in comparison to one another. Therefore, this retrospective evaluation for superiority was performed. The primary outcome was urinary stress incontinence, and secondary outcomes were operative factors. There were 185 PC slings, but 12 were excluded because of urethral plications. There were 50 RPCF procedures, but 3 were excluded because of urethral plications. Finally, there were 32 cases with both PC sling and RPCF procedures. All groups demonstrated a higher than expected fistula repair rate with negative dye tests in 84% of the PC sling group, 89.9% in the RPCF group, and 93.8% in the RPCF and PC groups. There were no statistically significant differences found in continence status between the three groups. Of those who underwent PC slings, 49% were found to have residual stress incontinence. Of those who underwent RPCF, 47.8% had stress incontinence. Of those with both techniques, 43.8% had residual stress incontinence. Pad weight was not significantly different between the groups. As there is no statistically significant difference, we cannot recommend one procedure over the other as an anti-incontinence procedure. The use of both simultaneously is worth investigating.

## 1. Introduction

**Figure 1:**
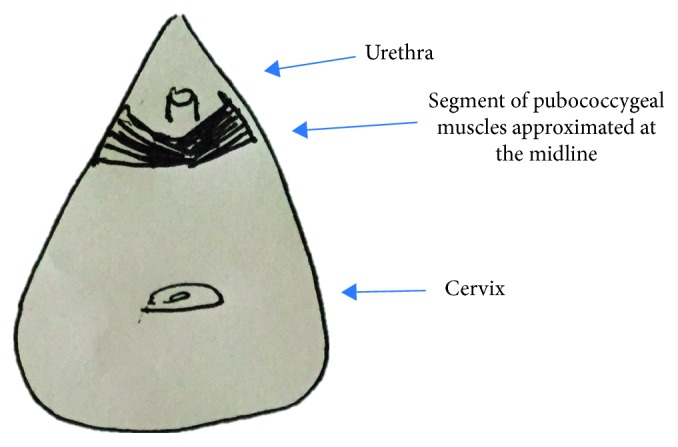
Pubococcygeal (PC) sling.

Residual urinary incontinence is a challenge in obstetric fistula surgery. Despite the successful closure of the vesicovaginal fistula, the urethra no longer functions properly. Many patients leak so severely that they do not consider themselves healed [1, 2]. Therefore, multiple interventions to create a barrier to the outpouring of urine have been attempted. Browning describes a fibromuscular vaginal sling using the pubococcygeal (PC) muscle with significant reduction in residual incontinence (Figure 1) [3]. He documents a stress incontinence rate of 39% (*n* = 272) compared to a previous rate without the sling for high-risk patients of 55% [4]. To date, no other author has documented their residual incontinence rate when incorporating the PC sling or compared it to another technique.

**Figure 2:**
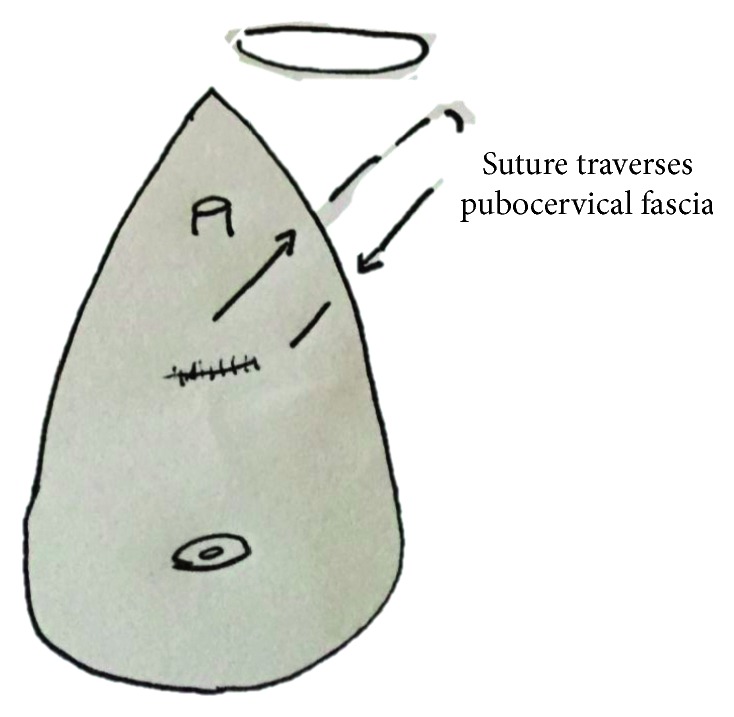
Placement of suture for refixation of pubocervical fascia (RPCF). This is done bilaterally.

Other surgeons utilize a technique called refixation of the pubocervical fascia (RPCF) in similar circumstances to those in which a PC sling might be used. That is, when the urethra is short or weakened and the fistula repair might compromise the urethra by applying caudal traction [5]. It is sometimes combined with a urethral plication or urethralization [5]. The pubocervical fascia originates at the arcus tendineus on the pelvic side wall and supports the urethra, the urethrovesical junction, and the bladder base. An RPCF is carried out by placing a large delayed-absorbable suture in an anterior to posterior fashion on bilateral pelvic side walls where the PC fascia may be weakened (Figure 2). The procedure radically plicates the distal bladder and is thought to restore normal anatomy [6].

At our center, both techniques are commonly used by both visiting and resident surgeons and at times are used concurrently. This study's purpose is to identify the benefit of one over the other or the benefit of both used together.

## 2. Materials and Methods

This is a retrospective review, which is part of a larger study examining the outcomes of obstetric fistula surgical repairs at the Fistula Care Center in Lilongwe, Malawi. All participants gave oral and written consent to be included in the study. Medical charts entered into a RedCap Database were collected for any patient who received a PC sling and/or a RPCF at the time of vesicovaginal fistula repair. Patients with any other urethral plication or similar procedure were excluded as not all cases included plication. All charts were double entered by two research assistants and cleaned by a physician to ensure clinical accuracy.

The research questions were as follows: Is the proportion of stress incontinence the same in each group? What is the rate of stress incontinence (with 95% CI) in those who have the PC sling, RPCF, or both? The primary outcome was continent status with one-hour pad weight as the objective measure to complement the clinical diagnosis. After the dye test to assess for fistula healing, the urinary catheter was removed and patients were asked to cough to assess for stress urinary incontinence. They were then given a sanitary pad to wear for one hour. The pad was then weighed. Secondary outcomes were OR time, estimated blood loss, and OR complications.

Power analysis was performed based on published findings of PC slings resulting in urethral/stress incontinence for 39% of high-risk patients (Browning). To be sufficiently powered at 80%, at least 59 cases in the RPCF and PC sling arm were needed. The continuous variables examined did not show a normal distribution displayed by Q-Q plots. Therefore, nonparametric tests were performed (Kruskal–Wallis) to compare the means. Categorical variables were compared with the chi-squared test.

## 3. Results

A total of 1,550 total procedures were done from 2011 to October 2017. Mean operating time per procedure was 72.4 minutes (SD: 45.9), and mean estimated blood loss was 107.4 mL (SD: 129.3). Of these cases, 185 underwent PC slings, but 12 were excluded because of urethral plications. There were 50 RPCF procedures, but 3 were excluded because of urethral plications. Finally, there were 32 cases with both PC sling and RPCF procedures. Therefore, the PC and RPCF combination arm was underpowered to detect a significant difference between the groups.

All groups demonstrated a higher than expected fistula repair rate given the higher complexity of those undergoing RPCF and/or PC sling, with negative dye tests in 84% of the PC sling group, 89.9% in the RPCF group, and 93.8% in the RPCF and PC group (Table 1). There is a trend towards negative dye tests with the combination of the RPCF and PC. However, there were no statistically significant differences found in continence status between the three groups. Of those who underwent PC slings, 49% were found to have residual stress incontinence. Of those who underwent RPCF, 47.8% had stress incontinence. Of those with both techniques, 43.8% had residual stress incontinence. Pad weight was not significantly different between the groups.

None of the continuous variables displayed a normal distribution. Therefore, the statistics presented in Table 1 are nonparametric for all continuous variables. The chi squared test was performed on all categorical variables. The following were compared: PC sling to RPCF, PC sling to the combination of PC sling and RPCF, and RPCF to the combination of PC sling and RPCF. Comparing the continence status did show a significant difference when comparing with low capacity incontinence, which was primarily due to the difference seen between the PC sling and the RPCF arms (greater proportion of low-capacity incontinence in the RPCF arm). All other forms of incontinence and residual fistula were not different amongst the groups. The most common type of fistulas in which a PC and/or an RPCF were done were Goh classes 3 and 4 (Table 2). By comparing Goh classes 1 and 2 (where the fistula is >2.5 cm distance to the external urethral orifice) and Goh classes 3 and 4 (where the fistula is ≤2.5 cm from the external urethral orifice), those with Goh classes 3 and 4 are statistically significantly more likely to have stress incontinence and a heavier pad weight (Table 3).

Regarding secondary outcomes, there was no significant difference in the procedures comparing the operative time, estimated blood loss, or postoperative complications (Table 1).

## 4. Discussion

Our results demonstrate no statistically significant difference between the PC sling and RPCF. Although both procedures are often done to prevent residual incontinence for high-risk patients, several patients who received each procedure were classified as Goh 1 or 2, which reflects a normal urethral length. When the individuals with a shorter urethral length (Goh classes 3 and 4), a known risk factor for residual stress incontinence, are compared to those with a normal length, a statistically significant larger proportion have stress incontinence and a heavier pad weight (Table 3). Also, low capacity as a cause of urethral incontinence was greater in the RPCF group. This may reflect a patient with a poorer prognosis and a tendency on the part of the surgeon to employ the RPCF in these patients, although this cannot be confirmed with the data.

As no difference in secondary patient care outcomes such as operative times, estimated blood loss, and complications resulted between groups, neither procedure appears superior to the other. Subjectively, one advantage of the RPCF is that it seems to assist in gaining hemostasis after difficult dissections in the paravesical space, whereas the PC sling often causes bleeding during the dissection. However, the difference in total estimated blood loss between the two is not significantly different. Several of our patients who have had a RPCF placed complain of prolonged tenderness at the area of the suture knots postoperatively. However, this was not systematically assessed in all patients and may be ameliorated with a different suture type. Although this is a subjective disadvantage, if there is no superiority of the RPCF over the PC sling, it could be beneficial to opt for the PC sling to avoid the possible discomfort. Otherwise, if the discomfort is not significant when assessed prospectively, the use of both the PC sling and the RPCF may be beneficial together and is worth investigating.

Limitations of the study were that it was not sufficiently powered to determine if a combination of procedures is superior to the individual procedures. The retrospective nature of the study is also a limitation.

## 5. Conclusions

No other studies have been conducted on this topic to our knowledge. As overall 10–15% of women with OF and 50% of women with Goh classes 3 and 4 OF experience residual stress incontinence after OF repair, we are constantly pursuing ways to prevent stress incontinence [7, 8]. It appears that both the PC sling and the RPCF are comparable options, but many women will still experience urinary leakage. A larger sample size and a prospective study would be useful to determine if a combination of the PC and RPCF is superior to one or the other. If not, new methods to prevent incontinence are needed to reduce the number of women experiencing residual incontinence after vesicovaginal fistula repair.

## Figures and Tables

**Table 1:** Continent status across groups.

		Procedure		
	PC sling mean (SD)	RPCF	*P* value	PC sling + RPCF
Total	176	73		32

Operating time (min)	92.8 (44.9)	89.0 (43.1)	0.6508	92.3 (37.0)
	86.1–99.6	78.6–99.3		79.0–105.6

Estimated blood loss (cc)	130 (136.7)	123.7 (185.5)	0.3960	132.7 (138.8)
	109.3–150.7	76.6–170.8		82.6–182.7

Postoperative complications			0.595	
(i) Bleeding	1 (0.06)	1 (1.4)		0 (0.0)
(ii) Suspected infection	3 (1.7)	2 (2.7)		0 (0.0)
(iii) Urinary retention	0 (0.0)	1 (1.4)		0 (0.0)
(iv) No complications	172 (97.7)	69 (94.5)		32 (100.0)

Dye test results	169	69	0.221	32
(i) Positive	27 (16.0)	7 (10.1)		2 (6.7)
(ii) Negative	142 (**84.0**)	62 (**89.9**)		30 (**93.8**)

Pad weight (g)	14.7 (20.1)	20.7 (25.4)	0.2143	14.8 (19.7)
	11.7–17.8	14.8–26.6		7.7–21.9

Clinical impression of incontinence status	157	67	0.515	32
(i) Continent	55 (35.0)	18 (26.9)		13 (40.6)
(ii) Stress	74 (**47.1**)	32 (**47.8**)		14 (**43.8**)
(iii) Urge	2 (1.3)	1 (1.5)		0 (0.0)
(iv) Low capacity	6 (3.8)	8 (11.9)		2 (6.3)
(v) Residual fistula	20 (12.7)	8 (11.9)		3 (9.4)

**Table 2:** Fistula classification across groups.

	PC sling	RPCF	PC + RPCF
Goh type	*N* = 169	*N* = 57	*N* = 30
1: distal edge of the fistula >3 cm from the EUO	5 (2.9)	1 (1.7)	1 (3.3)
2: 2.5–3 cm from the EUO	20 (11.8)	8 (14.0)	2 (6.7)
3: 1.5 ≤ 2.5 cm from the EUO	98 (58.0)	28 (49.1)	13 (43.3)
4: <1.5 cm from the EUO	46 (27.2)	20 (35.1)	14 (46.7)

A: size <1.5	26 (15.3)	6 (10.9)	2 (6.5)
B: 1.5–3 cm	74 (43.5)	24 (43.6)	7 (22.6)
C: >3 cm	70 (41.2)	25 (45.5)	22 (71.0)

(i) No or mild fibrosis	24 (14.2)	4 (7.4)	0 (0)
(ii) Moderate or severe fibrosis and/or reduced vaginal length	22 (13.0)	4 (7.4)	1 (3.2)
(iii) Special circumstances and repeat procedure	123 (72.8)	46 (85)	30 (96.8)

**Table 3:** Goh classification of those with stress incontinence.

	Stress incontinence		Continent	Pad weight mean (SD)	
Goh classes 1 and 2	9 (8.1)	*P*=0.007	22 (21.2)	7.5 (17.4)	*P*=0.0076
				1.7–13.3	
Goh classes 3 and 4	102 (91.9)		82 (78.8)	17.7 (22.0)	
				14.8–20.6	
